# Endoscopic-assisted, minimally invasive versus sternotomy total arterial multivessel bypass grafting

**DOI:** 10.1093/icvts/ivae187

**Published:** 2024-11-14

**Authors:** De Qing Görtzen, Fleur Sampon, Naomi Timmermans, Joost Ter Woorst, Ferdi Akca

**Affiliations:** Department of Cardiothoracic Surgery, Catharina Hospital, Eindhoven, Netherlands; Department of Cardiothoracic Surgery, Catharina Hospital, Eindhoven, Netherlands; Department of Cardiothoracic Surgery, Catharina Hospital, Eindhoven, Netherlands; Department of Cardiothoracic Surgery, Catharina Hospital, Eindhoven, Netherlands; Department of Cardiothoracic Surgery, Catharina Hospital, Eindhoven, Netherlands

**Keywords:** Endo-CAB, Minimally invasive, Coronary surgery, Thoracoscopic left internal mammary artery harvesting, Sternotomy, Textbook outcome

## Abstract

**OBJECTIVES:**

This single-centre study compared the perioperative outcomes after total arterial multivessel revascularization through endoscopic-assisted, minimally invasive surgery compared to a conventional sternotomy approach.

**METHODS:**

In this retrospective, propensity score-matched (PSM) cohort study, a total of 740 patients were analysed [endoscopic coronary artery bypass grafting (Endo-CAB), *N* = 92; Sternotomy, *N* = 648]. After PSM (1:2 ratio), 73 Endo-CAB and 137 sternotomy patients were compared with an equal number of distal anastomoses (Endo-CAB 2.3 versus Sternotomy 2.4 anastomoses per patient, *P* = 0.082). We used ‘textbook outcome’ as a patient-orientated outcome measure, defined as the absence of 30-day mortality, re-exploration for bleeding, postoperative ischaemia, cardiac tamponade, cerebrovascular events, wound infection, new onset arrhythmias, pneumonia, placement of chest drains and prolonged hospital stay (>7 days).

**RESULTS:**

Patients undergoing Endo-CAB had significantly more often a textbook outcome compared to the sternotomy group (78.1% vs 59.1%, *P* = 0.009). Endo-CAB patients had shorter hospital stay (4.0 vs 6.0 days, *P* < 0.001), less postoperative blood loss (360 vs 490 ml, *P* < 0.001) and a significant reduction of new onset postoperative atrial fibrillation (5.5% vs 17.5%, *P* = 0.015). Other postoperative outcomes were comparable for both groups.

**CONCLUSIONS:**

Total arterial Endo-CAB demonstrates excellent postoperative outcomes compared to a sternotomy approach for multivessel coronary artery disease. These findings provide a strong basis for further expanding the multivessel Endo-CAB programme.

## Graphical Abstract

**Figure:**
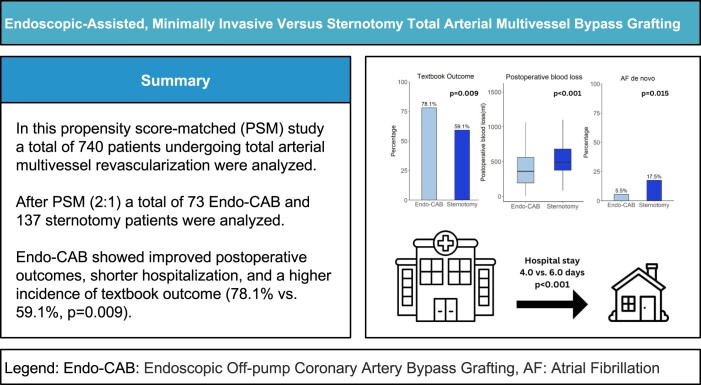

## INTRODUCTION

Surgical approaches and techniques for coronary revascularization have seen constant innovation since the 1st coronary artery bypass grafting (CABG) was performed in the early 1960s [1]. Minimally invasive revascularization techniques have gained increasing popularity in recent years [2]. For single-vessel surgical revascularization of the left anterior descending, a minimally invasive approach is performed in many centres worldwide with a variety of techniques [3–9]. However, minimally invasive multivessel total arterial bypass grafting is less often performed, and data comparing this technique to a sternotomy approach are scarce.

Off-pump coronary surgery allows complete revascularization on a beating heart without the need for cardiopulmonary bypass (CPB). This offers a reduced stroke risk when the aorta is not manipulated (so-called ‘anaortic approach’) and reduces the invasiveness of coronary bypass surgery [10–12]. This can be achieved with a minimally invasive approach avoiding a sternotomy, although this procedure requires a different surgical dexterity compared to the conventional arrested-heart CABG [9, 10, 13–15]. Also, endoscopic harvesting of the arterial conduits (left and right mammary arteries) can be technically challenging and comes with a steep learning curve [9, 16]. The ideal coronary revascularization surgery would be a minimally invasive, off-pump anaortic procedure using complete arterial revascularization to maximize both the short- and long-term benefits for the patient.

In this study, our objective was to evaluate if endoscopic-assisted off-pump multi-arterial bypass grafting [endoscopic coronary artery bypass grafting (Endo-CAB)] offers improved perioperative outcomes compared to a sternotomy approach. Based on our previous comparative propensity-matched study for single-vessel procedures, we hypothesized a similar effect for multivessel revascularization [17].

## MATERIALS AND METHODS

### Ethical statement

Written informed consent was obtained for the procedures from all patients. The study was approved by the institutional review board (registration number W23.029).

### Patient inclusion

This is a single-centre study conducted at Catharina Hospital in Eindhoven in which all patients who underwent complete arterial bypass grafting for multivessel disease between December 2018 and March 2024 were reviewed. Patients underwent either sternotomy on-pump bypass grafting (CABG, *N* = 270), sternotomy off-pump bypass grafting [off-pump coronary artery bypass grafting (OPCAB), *N* = 378] or endoscopic off-pump bypass grafting (Endo-CAB, *N* = 92). The surgical approach was determined based on the preference of the surgeon. All data were gathered and analysed retrospectively. Only primary revascularization procedures were included, and redo procedures were excluded. A database of baseline characteristics was created for OPCAB and CABG patients, and these patients were combined into the ‘sternotomy group’. Differences in outcome between sternotomy on- and off-pump surgeries were not expected with the number of included patients and low operative risk. To address the effect of selection bias and other confounding factors, the sternotomy group (*N* = 648) and Endo-CAB group (*N* = 92) were propensity-matched based on baseline characteristics (sex, age, height, weight, body mass index, body surface area, renal impairment, preoperative estimated glomerular filtration rate, level of urgency) and number of distal anastomoses, with a 2:1 matching ratio. The number of distal anastomoses was chosen as a matching covariate to achieve 2 comparable groups, as the required number of grafts affects the treatment allocation of patients in daily practice (e.g. patients requiring 2 grafts were more likely planned for a minimally invasive approach since introduction). A 2:1 propensity match was performed (Sternotomy, *N* = 137; Endo-CAB, *N* = 73).

Contraindications for performing an Endo-CAB procedure were haemodynamic instability at presentation, morbid obesity (body mass index >40), severe chest wall deformities, previous cardiac or left-sided thoracic surgery, severe pulmonary dysfunction, severe left ventricular dysfunction (LVEF <30%) or haemodynamically significant left subclavian stenosis. A contraindication for sternotomy on-pump CABG was severe calcification of the ascending aorta in which case, we would perform off-pump CABG by a dedicated surgeon. For sternotomy off-pump CABG, there were no contraindications, besides salvage surgery.

#### Study end-points

The main end-point of our study was to compare procedural and perioperative 30-day outcomes between multi-arterial sternotomy and Endo-CAB patients. Besides comparison of the separate end-points, we compared the ‘textbook outcome’ (TO) between the groups. This is a composite quality measure that encompasses multiple postoperative end-points representing the ideal ‘textbook’ hospitalization for complex surgical procedures [17–20]. We defined TO as the absence of (i) 30-day mortality, (ii) re-exploration for bleeding, (iii) postoperative ischaemia (requiring either graft revision or acute percutaneous intervention), (iv) cardiac tamponade, (v) cerebrovascular events, (vi) wound infection requiring antibiotics or intervention, (vii) new-onset atrial fibrillation requiring medical therapy, (viii) pneumonia requiring antibiotics, (ix) placement of chest drains within 30 days and (x) a hospital stay more than 7 days.

Postoperative parameters and complications were recorded during hospital admission and until 30 days of follow-up. If a postoperative patient was transferred to the referring hospital after a few days for further recovery, all discharge letters from these hospitals were collected to ensure a complete data set. Furthermore, all patients had a visit on our outpatient clinic 2–4 weeks after discharge.

### Endoscopic coronary artery bypass grafting procedure

The method of endoscopic harvesting the left and right internal mammary arteries has previously been described [17]. The patient is in a supine position, and to provide better access during the procedure, a pillow is placed underneath the left scapula to raise the left hemithorax. Standard arterial and central venous lines, along with an endotracheal tube, are placed before the start of the procedure. A selective endobronchial blocker (Teleflex, Wayne, PA, USA) is placed through the endotracheal tube to allow unilateral ventilation during the grafting process. The groins are marked and prepared for femoral cannulation in the event of haemodynamic instability. If the radial artery is being harvested endoscopically using the Vasoview Hemopro system (Getinge, Gothenburg, Sweden), the right arm is placed 90 degrees angle from the thorax and allows simultaneous harvesting of the graft conduits. Additionally, transoesophageal echocardiography is used to assess left ventricular and valve function.

**Figure 1:**
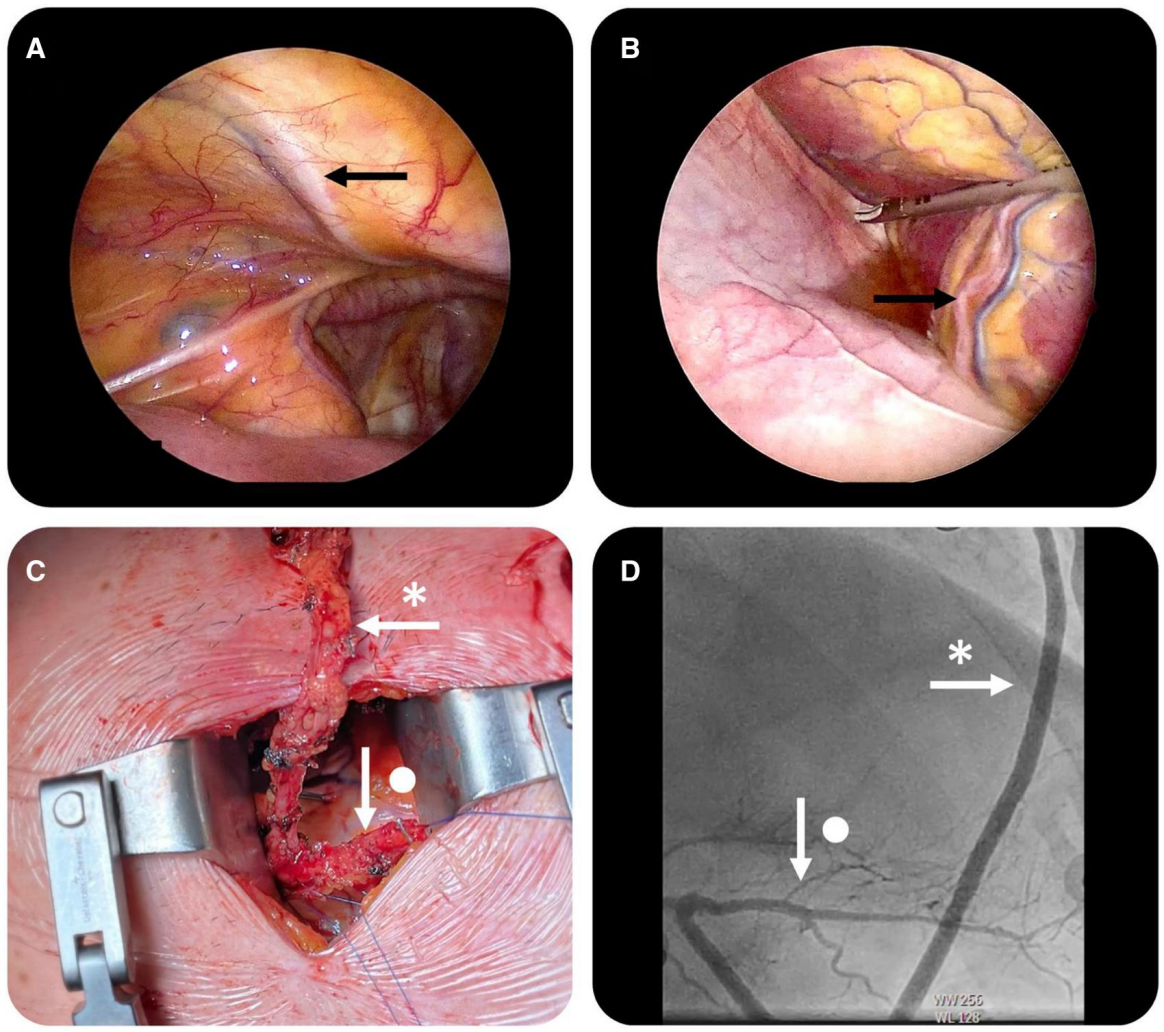
Images of Endo-CAB surgery illustrating (**A**) the endoscopic view of the LIMA—marked by a black arrow, (**B**) the right descending coronary artery marked by the black arrow, (**C**) shows the Y-graft configuration through the mini-thoracotomy of the radial artery marked by the star arrow on the LIMA marked by the dot arrow and (**D**) the angiographic result of the radial artery (star) on the right posterior descending coronary artery (dot). Endo-CAB: endoscopic coronary artery bypass grafting; LIMA: left internal mammary artery.

Three small incisions are made on the left side of the thorax, 5-mm endoscopic ports are placed and a capnothorax is created. A 5-mm zero-degree two-dimensional camera (Karl Storz GmbH, Tuttlingen, Germany) and standard long shafted video-assisted thoracic surgery instruments and the Ligasure Maryland device (Medtronic, Dublin, Ireland) are introduced. Before mammary artery harvesting, the pericardium is opened anteriorly to the left phrenic nerve to identify and investigate coronary targets (Fig. 1). Once all conduits are harvested, heparin is administered intravenously to achieve an activated clotting time of over 300 s.

A 5 cm mini-thoracotomy is created depending on the patient’s specific anatomy in the 3rd–5th intercostal space. During multi-arterial procedures, various graft configurations can be used depending on the used conduits and the location of the coronary targets. *In situ* bilateral mammary artery, or Y- or T-composite graft configurations with the radial or free right internal mammary can be used. To position the heart during multi-arterial Endo-CAB procedures, a heart-positioning device (Starfish Evo, Medtronic) and epicardial stabilizer (Octopus Nuvo, Medtronic) are used as described earlier by colleagues [3, 6, 21].

After the grafting process was complete, protamine was administered. The graft function was assessed using transit-time flow measurement (Medistim Vascular transit-time flow measurement, Medistim, Oslo, Norway). If graft flow was sufficient, a 28-Fr chest tube was placed in the left pleural cavity through 1 of the 3 previously made incisions for left internal mammary artery harvest. Ropivacaine (10 mg/ml) was administered at 3 intercostal levels with a total dose of 3 mg/kg and then the skin was closed.

### Sternotomy (coronary artery bypass grafting and off-pump coronary artery bypass grafting)

A full median sternotomy was performed during the multi-arterial CABG and OPCAB procedures. The mammary artery (left and/or right) was harvested under direct visualization, and the radial artery was harvested using open or endoscopic techniques. For CABG procedures, CPB was used and the heart was arrested using blood cardioplegia for the distal anastomosis. For the OPCAB procedure, bypass grafts were placed on the beating heart without CPB using epicardial stabilization (Octopus, Medtronic, Dublin, Ireland). Graft configurations were either composite Y-/T-grafts, bilateral mammary artery *in situ* grafts or a proximal graft on the ascending aorta. Subsequently, 2 chest tubes (26- and 28-Fr) were introduced into the retrosternal, pericardial, or pleural space. The sternum was closed using sternal wires.

### Follow-up

Standard follow-up appointments were scheduled after hospital discharge. Endo-CAB patients were scheduled for follow-up at 2 weeks, and OPCAB and CABG patients at 6 weeks, according to our standard in-hospital protocol. Additional hospital visits, including visits to the emergency department or readmissions within 30 days after discharge, were recorded and analysed.

### Statistical methods section

Data were analysed using JASP 0.18.1.0 (Universiteit van Amsterdam, The Netherlands) to extract descriptive, box plots and Q–Q plots for outliers per continuous variable. Categorical variables are presented as frequency and percentage. Continuous variables are presented as mean and standard deviation if normally distributed, and median and interquartile range if non-normally distributed as per graphical analysis through the Q–Q plots. If any outliers were identified, they were excluded from the propensity score matching (PSM) and further analysis. Furthermore, the Mann–Whitney *U*-test was used to compare the data. Where appropriate, a chi-square test or Fisher’s exact test was performed using RStudio version 4.4.0 (R Core Team, Boston, USA). PSM was used to adjust for differences in baseline characteristics and the number of distal anastomoses. At baseline, we identified differences in the unmatched group using Mann–Whitney *U* analyses. The variables for PSM were selected based on baseline differences identified in the unmatched cohorts and other potential confounding variables known from the literature. Furthermore, the standardized mean differences for each variable before and after matching were reported. The groups were matched on: sex, age, height, weight, body mass index, body surface area, renal impairment, preoperative estimated glomerular filtration rate, level of urgency and number of distal anastomoses. Cases were matched in a 2:1 ratio (Sternotomy: Endo-CAB) using propensity scores, with a calliper of 0.1 standard deviations from the logit of the propensity score, and without replacement. The chosen calliper of 0.1 balances the reduction of bias between the cohorts while maintaining a sufficient sample size for further analysis. After PSM, a standardized difference of 0.1 or less was considered an ideal balance, while 0.1–0.2 was deemed acceptable. The matching process aimed to achieve a reduction in overall standardized mean differences in the matched group. Cox proportional hazard analyses were performed to evaluate the hazard ratio for postoperative outcomes. The validity of the proportional hazard model assumption was assessed using Schoenfeld residuals through both a global test and individual tests for each covariate in the model, with no violations of the assumption detected.

## RESULTS

### Patient demographics

**Figure 2:**
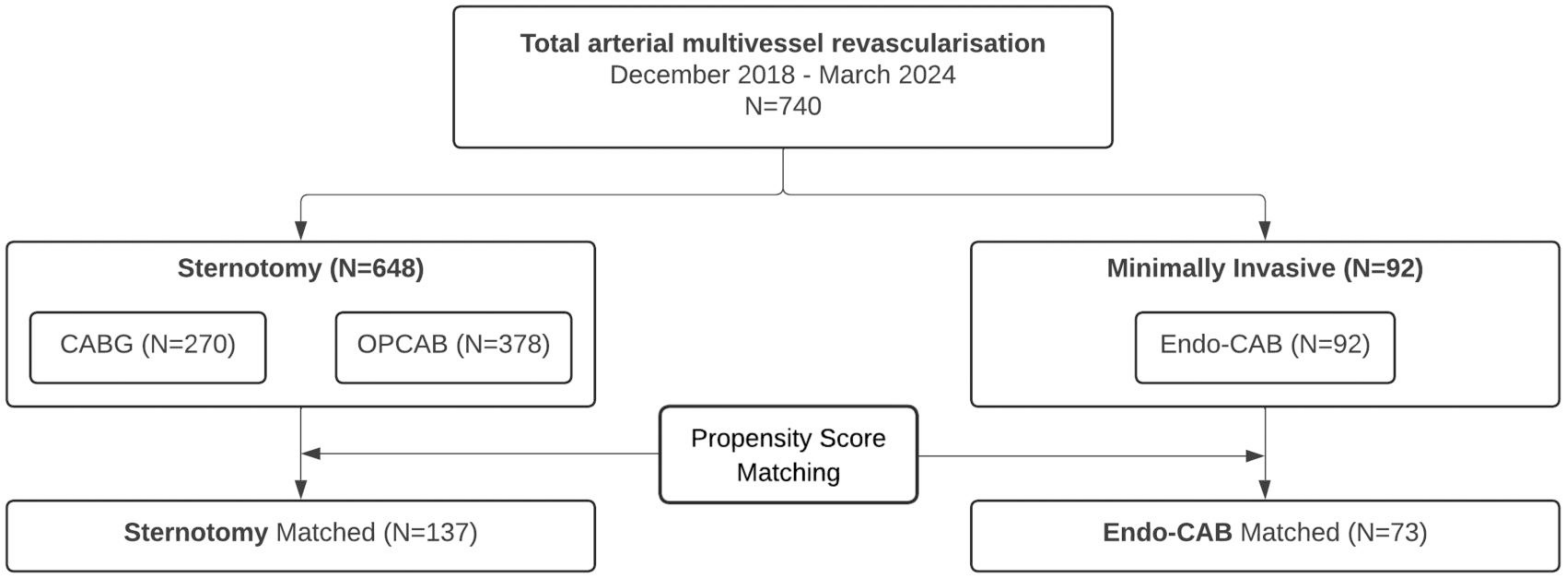
Flowchart of patient selection. CABG: coronary artery bypass grafting; Endo-CAB: endoscopic coronary artery bypass grafting; OPCAB: off-pump coronary artery bypass grafting.

**Figure 3:**
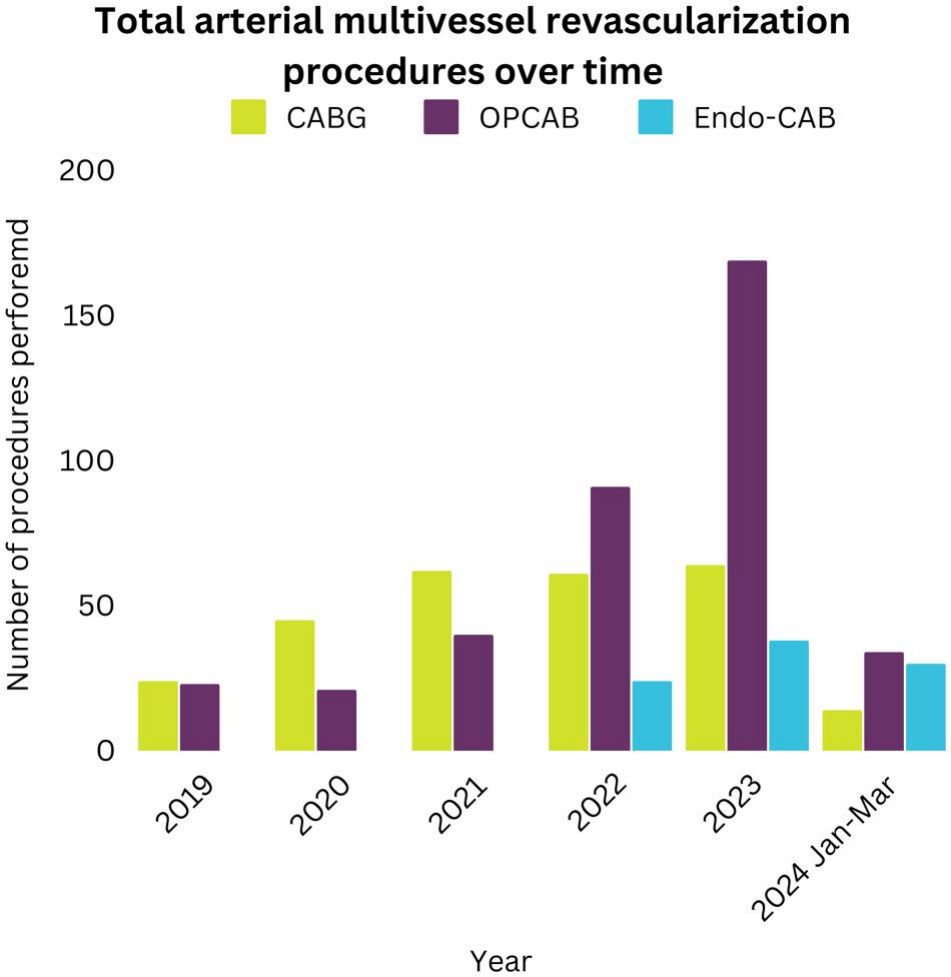
Distribution of the total arterial revascularization procedures over the years. CABG: coronary artery bypass grafting; Endo-CAB: endoscopic coronary artery bypass grafting; OPCAB: off-pump coronary artery bypass grafting.

A total of 740 patients underwent total arterial multivessel bypass grafting. After PSM based on the baseline characteristics and the number of distal anastomoses, a total of 210 patients were analysed (73 Endo-CAB versus 137 Sternotomy) (Fig. 2). From all patients, complete 30-day follow-up and all perioperative data were available. Demographic characteristics before and after matching are presented in Table 1. The mean age of the population was 62.4 ± 9.0 years and 88% were men. After propensity matching, all baseline characteristics were comparable between both groups. The distribution of the different procedure types over the years is presented in Fig. 3.

**Table 1:** Demographics–Sternotomy–Endo-CAB

	Unmatched					Propensity matched			
	Overall (*N* = 740)	Sternotomy (*N* = 648)	Endo-CAB (*N* = 92)	*P*-value	SMD	Sternotomy (*N* = 137)	Endo-CAB (*N* = 73)	*P*-value	SMD
Sex (men)	651 (88.0)	567 (87.5)	84 (91.3)	0.294	0.134	122 (89.1)	67(91.8)	0.533	0.016
Age (year)	62.4 ± 9.0	62.0 ± 8.9	66.0 [58.0–72.0]	**0.003**	0.344	62.9 ± 8.1	64.5 ± 9.5	0.190	0.121
Height (cm)	177 [172–182]	177 [172–182]	175 [171–180]	0.071	0.203	176 [170–181]	175 [172–180]	0.876	0.037
Weight (kg)	85.2 [77.8–95.9]	85.6 [78.0–96.2]	82.0 [74.0–90.0]	**0.010**	0.359	85.4 ± 12.9	84.5 ± 12.6	0.804	0.063
BMI (kg/m^2^)	27.1 [25.2–30.3]	27.1 [25.2–30.4]	27.1 [24.7–28.7]	0.088	0.301	27.7 ± 3.8	27.3 ± 3.4	0.737	0.049
BSA (m^2^)	2.03 [1.92–2.15]	2.04 [1.92–2.15]	1.99 [1.89–2.08]	**0.004**	0.324	2.03 [1.91–2.13]	2.01 [1.92–2.10]	0.590	0.067
Diabetes	143 (19.3)	128 (19.8)	15 (16.3)	0.434	0.091	24 (17.5)	12 (16.4)	0.845	0.003
Peripheral vascular disease	64 (8.6)	58 (9.0)	6 (6.5)	0.439	0.111	17 (12.4)	4 (5.5)	0.112	0.037
Atrial fibrillation	32 (4.3)	27 (4.2)	5 (5.4)	0.577	0.071	10 (7.3)	4 (5.5)	0.617	0.008
Previous cardiac surgery	4 (0.5)	4 (0.6)	0	1.000		0	0		
Recent MI	222 (30.0)	194 (29.9)	28 (30.4)	0.923	0.019	39 (28.5)	25 (34.2)	0.388	0.037
Prior PCI	194 (26.2)	161 (24.8)	29 (31.5)	0.217	0.128	46 (33.6)	23 (31.5)	0.763	0.023
Normal LV function (≥50%)	600 (81.1)	520 (80.2)	76 (82.6)	0.632	0.071	114 (83.2)	59 (80.8)	0.808	0.062
COPD	31 (4.2)	26 (4.0)	5 (5.4)	0.525	0.057	8 (5.8)	5 (6.8)	0.775	0.014
Pulmonary Hypertension	9 (1.2)	7 (1.1)	2 (2.2)	0.372	0.072	1 (0.7)	2 (2.7)	0.246	0.001
CVA	29 (3.9)	23 (3.5)	6 (6.5)	0.170	0.115	4 (2.9)	5 (6.8)	0.183	0.036
Renal impairment	88 (12.0)	83 (12.8)	5 (5.4)	**0.041**	0.317	9 (6.6)	5 (6.8)	0.941	0.020
Dialysis	4 (0.5)	4 (0.6)	0	1.000		1 (0.7)	0	1.000	
Level of urgency				**<0.001**	0.381			0.386	0.070
Elective	263 (35.3)	214 (33.0)	47 (51.1)			52 (38.0)	32 (43.8)		
Urgent	450 (60.8)	406 (62.7)	44 (47.8)			82 (59.9)	40 (54.8)		
Emergency	26 (3.5)	25 (3.9)	1 (1.1)			3 (2.2)	1 (1.4)		
Salvage	1 (0.1)	1 (1.1)	0			0	0		
Euroscore II	1.08 [0.79–1.63]	1.09 [0.79–1.66]	1.00 [0.80–1.61]	0.533	0.253	1.00 [0.77–1.74]	0.99 [0.76–1.60]	0.852	0.093
Preoperative haemoglobin (mmol/l)	9.0 [8.4–9.5]	9.0 [8.4–9.5]	9.1 [8.3–9.6]	0.942	0.052	8.9 [8.4–9.5]	9.1 [8.4–9.6]	0.694	0.082
Preoperative eGFR (ml/min/1.72 m^2^)	81 [70–90]	82 [70.0–90.0]	78 [68–89]	0.076	0.143	79 [71–90]	79 [71–89]	0.826	0.061
Average number of distal anastomoses	3.2	3.3	2.3	**<0.001**	2.364	2.4	2.3	0.082	0.089

BMI: body mass index; BSA: body surface area; COPD: chronic obstructive pulmonary disease; CVA: cerebral vascular event; eGFR: estimated glomerular filtration rate; Endo-CAB: endoscopic coronary artery bypass; LV: left ventricular function; MI: myocardial infarction; PCI: percutaneous coronary intervention; SMD: standardized mean difference. *P* values < 0.05 in bold.

### Procedural parameters

In Table 2, the procedural parameters are demonstrated. The mean number of distal anastomoses was 2.4 ± 0.5 for the entire study population. The left internal mammary artery was used in all patients for both sternotomy and Endo-CAB patients. As a 2nd arterial conduit, the radial artery was used in 51.1% during sternotomy procedures and in 76.7% during Endo-CAB. The right internal mammary was used in 50.4% and 21.9%, respectively. Most frequently, composite Y-graft configurations were made (Endo-CAB 83.6% versus Sternotomy 68.6%, *P* = 0.286). In 3 CABG patients, an OPCAB was performed due to severe calcifications of the aorta. In the Endo-CAB group, 2 patients (2.7%) had CPB support during the procedure (1 preplanned and 1 due to haemodynamic instability). Total procedural time was significantly longer for the Endo-CAB group compared to sternotomy procedures [198 (184–222) versus 164 (139–192) (min), *P* < 0.001].

**Table 2:** Procedural parameters–Sternotomy–Endo-CAB

	Unmatched				Propensity matched		
	Overall (*N* = 740)	Sternotomy (*N* = 648)	Endo-CAB (*N* = 92)	*P*-value	Sternotomy (*N* = 137)	Endo-CAB (*N* = 73)	*P*-value
Average number of distal anastomoses	3.2	3.3	2.3	**<0.001**	2.4	2.3	0.082
Number of distal anastomoses	2375	2168	207	**<0.001**			0.082
2	166 (22.4)	96 (14.8)	70 (76.1)		78 (56.9)	51 (69.9)	
3	319 (43.1)	298 (46.0)	21 (22.8)		59 (43.1)	21 (28.8)	
4	194 (26.2)	193 (29.8)	1 (1.1)		0	1 (1.4)	
5	56 (7.6)	56 (8.6)	0		0	0	
6	5 (0.7)	5 (0.8)	0		0	0	
Number of anastomoses with LIMA	969	873	96	**<0.001**			0.364
1	508 (68.6)	420 (65.0)	88 (95.6)		127 (92.7)	70 (95.9)	
2	229 (30.9)	225 (34.7)	4 (4.3)		10 (7.3)	3 (4.1)	
3	1 (0.1)	1 (0.2)	0		0	0	
Number of anastomoses with RIMA	615	586	29	**<0.001**			**<0.001**
1	128 (17.3)	103 (15.9)	25 (27.2)		53 (38.7)	14 (19.2)	
2	154 (20.8)	152 (23.5)	2 (2.2)		16 (11.7)	2 (2.7)	
3	53 (7.2)	53 (8.2)	0		0	0	
4	5 (0.7)	5 (0.8)	0		0	0	
Number of anastomoses with Radial Artery	791	709	82	0.315			**0.009**
1	110 (14.9)	66 (10.2)	44 (47.8)		37 (27.0)	37 (50.7)	
2	226 (30.5)	207 (31.9)	19 (20.7)		33 (24.1)	19 (26.0)	
3	67 (9.1)	67 (10.3)	0		0	0	
4	7 (0.9)	7 (1.1)	0		0	0	
Y-graft configuration	596 (80.5)	523 (80.7)	73 (79.3)	**0.022**	94 (68.6)	61 (83.6)	0.218
Bilateral internal mammary artery *in situ*	98 (13.2)	79 (12.2)	19 (20.7)	**0.025**	29 (21.2)	11 (15.1)	0.286
Aortic proximal anastomosis	50 (6.8)	50 (7.7)	0	1.000	16 (11.7)	0	1.000
Concomitant arrhythmia surgery	29 (3.9)	24 (3.7)	5 (5.4)	0.424	7 (5.1)	4 (5.5)	0.911
Hybrid revascularization	9 (1.2)		9 (9.8)			8 (11.0)	
Conversion to sternotomy	2 (0.3)		2 (0.3)			2 (2.7)	
On-pump to off-pump	13 (1.8)	13 (2.0)			3 (2.2)		
Off-pump to on-pump	9 (1.2)	7 (1.1)	2 (2.2)	0.372	0	2 (2.7)	
CPB assisted	271 (36.6)	270 (41.7)	2 (2.2)	**<0.001**	55 (40.2)	2 (2.7)	**<0.001**
ECC time (min)	79 [65–98]	79 [65–98]	49 [37–60]		61 [53–80]	49 [37–60]	0.351
X-time (min)	54 [44–71]	54 [44–71]			40 [34–53]		
Total operation time (hours)	186 [164–215]	184 [160–214]	197 [180–222]	**<0.001**	164 [139–192]	198 [184–222]	**<0.001**

AF: atrial fibrillation; CPB: cardiopulmonary bypass; ECC: extra corporeal circulation; LAA: left atrial appendage; LIMA: left internal mammary artery; RIMA: right internal mammary artery. *P* values < 0.05 in bold.

### Postoperative outcomes

Postoperative outcomes are displayed in Table 3. A TO occurred more frequently in the Endo-CAB group (78.1%) compared to the sternotomy group (59.1%, *P* = 0.009). Endo-CAB patients had shorter hospital stay (4.0 vs 6.0 days, *P* < 0.001), less postoperative blood loss (360 vs 490 ml, *P* < 0.001) and higher levels of haemoglobin at discharge (7.7 vs 7.0 mmol/l, *P* = 0.005). Postoperative ventilation time was similar for both groups (4.1 vs 4.0 h, *P* = 0.641). There was 1 (0.7%) case of hospital mortality case in the sternotomy group due to postoperative septic shock caused by gastrointestinal ischaemia and 1 (1.4%) case in the Endo-CAB group due to shock caused by postoperative stress response due to previously undiagnosed cancer metastases. Other postoperative outcomes were comparable for both procedures (Table 3). There were no strokes in the Endo-CAB group and 2 (1.5%) in the sternotomy group after off-pump CABG procedures. In the sternotomy group, 24 (17.5%) patients developed new-onset atrial fibrillation requiring anticoagulation compared to 4 (5.5%) Endo-CAB patients (*P* = 0.015).

**Table 3:** Postoperative outcomes–Sternotomy–Endo-CAB

	Unmatched				Propensity matched			
	Overall (*N* = 740)	Sternotomy (*N* = 648)	Endo-CAB (*N* = 92)	*P*-value	Sternotomy (*N* = 137)	Endo-CAB (*N* = 73)	*P*-value	Hazard ratio (95% CI)
Textbook outcome					81 (59.1)	57 (78.1)	**0.009**	1.39 [1.04–1.85]
Total hospital stay (days)					6.0 [5.0–7.0]	4.0 [3.0–5.0]	**<0.001**	0.97 [0.95–0.98]
In hospital mortality	6 (0.8)	5.0 (0.8)	1 (1.1)	0.754	1 (0.7)	1 (1.4)	0.655	1.25 [0.31–5.03]
Re-exploration bleeding	19 (2.6)	18 (2.8)	1 (1.1)	0.409	4 (2.9)	1 (1.4)	0.487	0.76 [0.31–1.84]
Ischaemia postoperative graft revision	9 (1.2)	9 (1.4)	0	1.000	1 (0.7)	0	1.000	0.44 [0.06–3.13]
Acute PCI	5 (0.7)	5 (0.8)	0	1.000	0	0		
Cerebrovascular accident	7 (0.9)	7 (1.1)	0	1.000	2 (1.5)	0	1.000	0.44 [0.11–1.76]
Tamponade during initial admission	5 (0.7)	5 (0.8)	0	1.000	2 (1.5)	0	1.000	0.44 [0.11–1.76]
Tamponade after discharge	11 (1.5)	10 (1.5)	1 (1.1)	0.736	4 (2.9)	1 (1.4)	0.487	0.76 [0.31–1.84]
New onset atrial fibrillation	87 (11.8)	81 (12.5)	6 (6.5)	0.096	24 (17.5)	4 (5.5)	**0.015**	0.63 [1.06–2.36]
Hospital-acquired pneumonia	27 (3.6)	23 (3.5)	4 (4.3)	0.703	9 (6.7)	4 (5.5)	0.758	0.93 [0.61–1.88]
Thoracentesis	15 (2.0)	12 (1.9)	3 (3.3)	0.370	2 (1.5)	3 (4.1)	0.233	1.42 [0.29–1.71]
Wound infection	14 (1.9)	14 (2.2)	0	1.000	3 (2.2)	0	1.000	0.43 [0.14–1.37]
Mediastinitis	4 (0.5)	4 (0.6)	0	1.000	0	0		
30-day readmission	37 (5.0)	35 (5.4)	2 (2.2)	0.184	6 (4.4)	2 (2.7)	0.558	0.83 [0.59–2.42]
Postoperative ventilation time (hours)	4.0 [3.1–5.3]	4.0 [3.0–5.3]	4.2 [3.1–5.3]	0.489	4.0 [3.1–5.1]	4.1 [3.0–5.3]	0.641	1.00 [1.00–1.01]
Postoperative blood loss (ml)	465 [350–676]	480 [364–696]	360 [198–585]	**<0.001**	490 [375–680]	360 [192–560]	**<0.001**	1.00 [1.00–1.00]
ICU admission time (days)	1.0 [0.5–1.0]	1.0 [0.5–1.0]	1.0 [0.5–1.0]	0.508	1.0 [0.5–1.0]	1.0 [0.5–1.0]	0.484	0.96 [0.84–1.09]
CK-MB maximum (ng/ml)	28 [21–38]	28 [22–39]	26 [19–35.3]	**0.039**	26 [20–35]	26 [19–33]	0.760	1.00 [1.00–1.00]
Discharge haemoglobin (mmol/l)	7.2 [6.4–8.0]	7.1 [6.4–7.9]	7.8 [6.9–7.9]	**<0.001**	7.0 [6.5–7.9]	7.7 [7.0–8.3]	**0.005**	1.15 [1.00–1.32]
Transfusion	106 (14.3)	96 (14.4)	10 (10.8)	0.313	22 (16.1)	8 (11.0)	0.317	0.85 [0.58–1.25]
Pulmonary embolism	7 (0.9)	6 (0.9)	1 (1.1)	0.883	1 (0.7)	1 (1.4)	0.655	1.25 [0.31–5.00]
Target vessel reintervention	19 (2.6)	15 (2.3)	4 (4.3)	0.249	4 (2.9)	4 (5.5)	0.359	1.26 [0.39–1.61]

CK-MB: creatine kinase-myoglobin binding; ECMO: extra corporeal membrane oxygenation; IABP: intra-aortic balloon pump; ICU: intensive care unit; LVAD: left ventricular assist device; PCI: percutaneous coronary intervention. *P* values < 0.05 in bold.

## DISCUSSION

### Study implications

In this study, we compared the outcomes of total arterial minimally invasive Endo-CAB with sternotomy surgery for multivessel coronary artery disease. Our propensity-matched data showed that total arterial Endo-CAB is associated with more frequent TO, representing the ideal ‘textbook hospitalization’. Patients undergoing multivessel Endo-CAB had a TO in 78.1% compared to 59.1% for sternotomy patients. We observed significantly less postoperative blood loss, lower incidence of new-onset atrial fibrillation and shorter hospital admission. Major postoperative complications, including the major adverse cardiac and cerebral events, were comparable for both techniques. The incidence of conversion to sternotomy (2.7%), support of CPB (2.7%) and mortality (1.4%) were low for total arterial Endo-CAB procedures. Performing concomitant endoscopic arrhythmia surgery was also feasible in addition to multivessel Endo-CAB. For selected patients, avoiding a sternotomy and offering a minimally invasive approach results in improved postoperative recovery. The endoscopic-assisted approach offers excellent views on the mammary artery during harvesting without the need for robotics, making it accessible to all cardiac surgery centres.

### Minimally invasive anaortic approach

Vallely *et al.* have previously discussed the superior outcomes and long-term survival benefits of CABG compared to percutaneous coronary intervention when it comes to multivessel revascularization. However, conventional on-pump CABG has an increased risk of stroke due to manipulation of the ascending aorta. The anaortic off-pump technique results in a decreased risk of postoperative complications [22]. Following the multivessel total arterial programme proposed by Gaudino *et al.* [23] starting from the off-pump approach, proceeding to the anaortic approach, and eventually offering a minimally invasive approach. It is important to focus on the technical challenges and core skills that need to be developed for each step [24]. Selecting the patient for right type of surgical strategy requires an individualized approach [23]. Minimally invasive multivessel revascularization can be performed both on-pump and off-pump. Babliak *et al*. have published the results of multivessel revascularization on the arrested heart with good results; however, this approach needs peripheral CPB and an arrested heart. Minimally invasive multivessel off-pump revascularization had been reported as well by several groups with good procedural outcomes using non-endoscopic techniques [3, 6, 7, 25, 26]. Experience with beating heart sternotomy surgery is essential to start a safe off-pump minimally invasive multivessel programme. The results of the MIST trial will also give more conclusive data on the outcome of minimally invasive compared to sternotomy CABG.

### Arterial revascularization

Multiple studies have suggested a total arterial revascularization strategy for multivessel disease as a superior strategy. Patients younger than 70 years with diabetes, atrial fibrillation and multivessel coronary artery disease benefit from a total arterial approach with improved long-term survival [12, 16, 24, 27–29]. The long-term patency of arterial conduit grafts has been investigated in multiple studies. Combining total arterial revascularization with an anaortic approach offers a reduced risk of stroke, mortality and myocardial infarction as demonstrated by the meta-analysis of Zhao *et al*. [30]. Tiwari *et al*. [28] showed that a minimally invasive total arterial procedure can be performed safely with good cosmetics and with a faster return to daily activities for the patient. Also, in our study, we observed an enhanced recovery of Endo-CAB patients with a shorter hospital admission and improved recovery compared to sternotomy surgery, despite our learning curve of this procedure. Careful patient selection and off-pump experience (also with single-vessel minimally invasive surgery) make the implementation of this technique feasible and safe. Therefore, the effect of the minimally invasive approach might even be underestimated.

### Study limitations

The study is a single-centre retrospective study with limited follow-up; therefore, generalizability remains the question. Furthermore, the angiographic patency of the grafts was limited to cases of hybrid revascularization or patients who underwent target vessel reinterventions for both groups without performing routine imaging follow-up.

## CONCLUSION

In this study, we observed that, in selected patients, the total arterial multivessel Endo-CAB has an excellent postoperative outcome with a shorter hospital stay and less postoperative blood loss compared to sternotomy revascularization. A TO occurred significantly more frequently in Endo-CAB patients compared to sternotomy patients. Other postoperative outcomes are comparable between the 2 techniques. These findings might encourage further expansion of minimally invasive multivessel off-pump revascularization.

## Data Availability

Data will be made available by the corresponding author upon reasonable request.

## ABBREVIATIONS

CABG: Coronary artery bypass grafting
CPB: Cardiopulmonary bypass
Endo-CAB: Endoscopic coronary artery bypass grafting
OPCAB: Off-pump coronary artery bypass grafting
PSM: Propensity score matching
TO: Textbook outcome
